# CRISPR/Cas9 Ablation of Integrated HIV-1 Accumulates Proviral DNA Circles with Reformed Long Terminal Repeats

**DOI:** 10.1128/JVI.01358-21

**Published:** 2021-11-09

**Authors:** Michele Lai, Eyal Maori, Paola Quaranta, Giulia Matteoli, Fabrizio Maggi, Marco Sgarbanti, Stefania Crucitta, Simone Pacini, Ombretta Turriziani, Guido Antonelli, Jonathan L. Heeney, Giulia Freer, Mauro Pistello

**Affiliations:** a Retrovirus Center, Virology Section, Department of Translational Research and New Technologies in Medicine and Surgery, University of Pisa, Pisa, Italy; b Laboratory of Viral Zoonotics, Department of Veterinary Medicine, University of Cambridge, Cambridge, United Kingdom; c Department of Medicine and Surgery, University of Insubria, Varese, Italy; d Virology Unit, Pisa University Hospital, Pisa, Italy; e National Institute of Health, Rome, Italy; f Pharmacology Unit, Department of Clinical and Experimental Medicine, University of Pisa, Pisa, Italy; g Hematology Unit, Department of Clinical and Experimental Medicine, University of Pisa, Pisa, Italy; h Laboratory of Virology, Department of Molecular Medicine, Sapienza University of Rome, Rome, Italy; i Pasteur Institute-Cenci Bolognetti Foundation, Department of Molecular Medicine, Sapienza University of Rome, Rome, Italy; Icahn School of Medicine at Mount Sinai

**Keywords:** CRISPR/Cas9, gene therapy, endonucleases, gene editing, HIV-1, latent reservoir, Tat, Rev, J-Lat, endonuclease, latent infection

## Abstract

Gene editing may be used to excise the human immunodeficiency virus type 1 (HIV-1) provirus from the host cell genome, possibly eradicating the infection. Here, using cells acutely or latently infected by HIV-1 and treated with long terminal repeat (LTR)-targeting CRISPR/Cas9, we show that the excised HIV-1 provirus persists for a few weeks and may rearrange in circular molecules. Although circular proviral DNA is naturally formed during HIV-1 replication, we observed that gene editing might increase proviral DNA circles with restored LTRs. These extrachromosomal elements were recovered and probed for residual activity through their transfection in uninfected cells. We discovered that they can be transcriptionally active in the presence of Tat and Rev. Although confirming that gene editing is a powerful tool to eradicate HIV-1 infection, this work highlights that, to achieve this goal, the LTRs must be cleaved in several pieces to avoid residual activity and minimize the risk of reintegration in the context of genomic instability, possibly caused by the off-target activity of Cas9.

**IMPORTANCE** The excision of HIV-1 provirus from the host cell genome has proven feasible *in vitro* and, to some extent, *in vivo*. Among the different approaches, CRISPR/Cas9 is the most promising tool for gene editing. The present study underlines the remarkable effectiveness of CRISPR/Cas9 in removing the HIV-1 provirus from infected cells and investigates the fate of the excised HIV-1 genome. This study demonstrates that the free provirus may persist in the cell after editing and in appropriate circumstances may reactivate. As an episome, it might be transcriptionally active, especially in the presence of Tat and Rev. The persistence of the HIV-1 episome was strongly decreased by gene editing with multiple targets. Although gene editing has the potential to eradicate HIV-1 infection, this work highlights a potential issue that warrants further investigation.

## INTRODUCTION

Highly active antiretroviral therapy (HAART) efficiently abates human immunodeficiency virus type 1 (HIV-1) replication and has transformed a deadly infection into a chronic illness. Unfortunately, HAART does not eradicate infection. By stalling viral replication, HAART halts HIV-1 spread to other cells but does not stop HIV-1 from persisting and reactivating when possible. Clustered regularly interspaced short palindromic repeats/Cas9 (CRISPR/Cas9), a technique that is changing paradigms and expectancies to cure genetic diseases (1), holds promise to provide a cure for HIV-1 as well (2). CRISPR/Cas9 can cut out the integrated HIV-1 genome (provirus) from the host cell genome (3) and has proven effective in eliminating, within certain limits, infection *in vitro* and in humanized mice (4).

The HIV-1 long terminal repeats (LTRs) comprise sequence domains recognized by cellular and viral proteins driving viral expression (5). Initial attempts to eliminate HIV-1 infection were performed using a single CRISPR/Cas9 guide RNA (gRNA) recognizing a sequence present in both LTRs of nearly all strains (3). This approach, while effective at curing some infected cells, was recently demonstrated to facilitate virus escape (6–8) through the ensuing nonhomologous end joining (NHEJ) repair mechanism (9). It has also been shown that CRISPR/Cas9 alone is not always sufficient to eliminate HIV-1 infection (4). At the moment, however, CRISPR/Cas9 is the most effective gene-editing method to excise HIV-1-infected cells, and it has proven safe for human cells and animal models, therefore exhibiting good prerequisites for clinical use (10–12).

The rationale of the present study is based on previous observations showing that most of the reverse-transcribed HIV-1 RNA genome (cDNA) produced during viral replication does not integrate (13, 14). Such unintegrated viral molecules are thought to either be destroyed or aid productive infection through the expression of several genes (15). Despite that, while these LTR circles are often considered dead ends of the viral life cycle (13, 14), we hypothesize that in the context of genomic instability caused by either on-target or off-target DNA cleavage, these elements might have a second chance of integrating through complementation or homology-directed repair (HDR) induced by gene editing (16). For this purpose, we used a single CRISPR/Cas9 guide RNA targeting both LTRs. Experiments were conducted in human embryonic kidney 293T cells bearing integrated, labeled HIV-1-pseudotyped vectors, and we then extended our observations to human T cell leukemia cells actively replicating HIV-1, as occurs during acute infection, or latently infected J-Lat cells, as observed in the asymptomatic phase. The results show that the excised provirus persists in the nucleus for a prolonged period of time; depending on the number of copies per cell, it closes as a single molecule (intramolecular) or with another (intermolecular), yielding circular elements that can, even if at a very low frequency, form complete LTRs again, thereby reducing the efficiency of HIV-1 eradication by gene editing. LTR circles generated by excised HIV-1 genomes exhibit LTRs that can respond to exogenous Tat and Rev and drive viral transcriptional activity again. Once LTRs are targeted with multiple guides, these elements lose their ability to be recovered by Tat and Rev, and more importantly, they cannot be active even if reintegrated through HDR.

## RESULTS

### CRISPR/Cas9 treatment efficiently excises the HIV-1 provirus.

**FIG 1:**
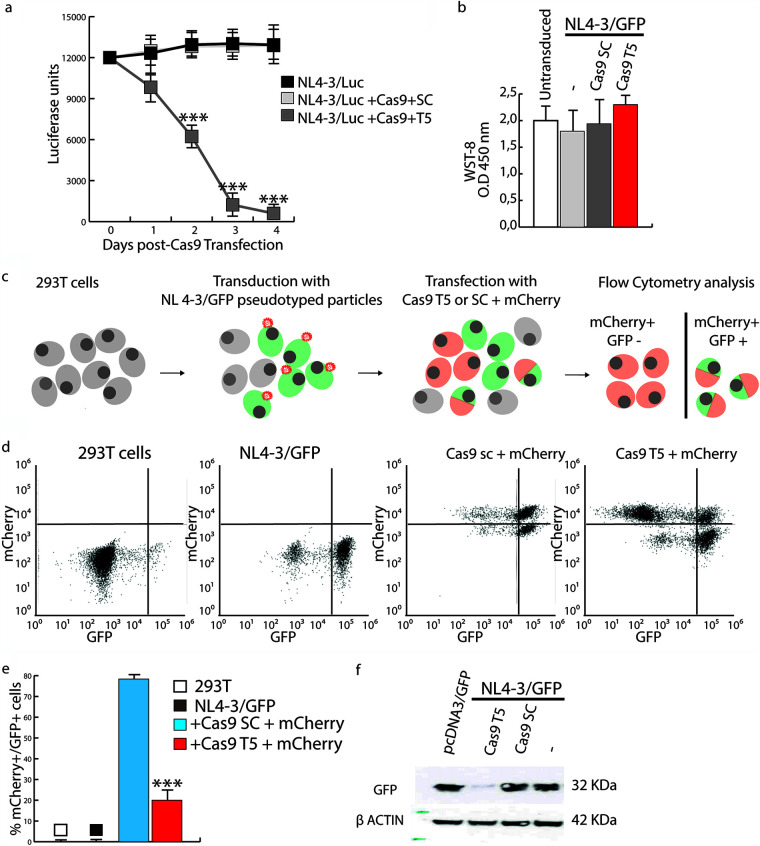
CRISPR/Cas9 efficiently cleaves the HIV-1 provirus in 293T cells transduced with NL4-3/GFP or NL4-3/Luc. (a) 293T cells that had been transduced with NL4-3/Luc pseudotyped with VSV-G were transfected with CRISPR/Cas9 and either scrambled (SC) or HIV-1-specific (T5) gRNAs and analyzed for Luc expression at days 0, 1, 2, 3, and 4. The decrease in Luc expression in T5 gRNA-treated cells compared to untreated or SC gRNA-treated cells reached statistical significance at day 2 (*P < *0.001). The standard deviation (SD) was calculated from three independent experiments. (b) Cell viability was assessed by WST-8 48 h after CRISPR/Cas9 transfection and showed no significant difference between cells transfected with NL4-3/Luc alone or in combination with Cas9 plus SC or T5 gRNAs and untreated cells. Shown are the means of data from three independent experiments with 6 technical replicates and SD. Statistical analyses were performed using Student’s *t* test. O.D, optical density. (c) Schematic of the flow cytometry analysis of GFP expression by 293T cells that either were not transduced or had been previously transduced with NL4-3/GFP pseudotyped with VSV-G and left as such (NL4-3/GFP) or then transfected with the SC or T5 gRNA-containing CRISPR/Cas9 mCherry plasmid. (d) A representative experiment of fluorescence-activated cell sorter (FACS) analysis of cells described above for panel c was performed at day 4 posttransfection and showed that T5 significantly decreased GFP expression by the provirus. (e) Statistical analysis of the values obtained in panel d. The means of data from three independent experiments with biological triplicates and SD are shown. Statistical analyses were performed using Student’s *t* test (***, *P *< 0.001) on mCherry^+^ cells (transfected by CRISPR/Cas9 only). (f) Western blot analysis of GFP protein levels at day 7 in lysates from 293T cells first transduced with NL4-3/GFP pseudotyped with VSV-G and then either left as such (−) or transfected with Cas9 plus SC or T5 gRNAs. A control of cells transfected with a GFP-encoding plasmid (pcDNA3/GFP) is included.

Human 293T cells were transduced with vesicular stomatitis virus G protein (VSV-G)-pseudotyped NL4-3/luciferase (Luc) lentiviral particles. The HIV-1 NL4-3-based construct expresses Luc under the control of the 5′ LTR but is defective in *env* and therefore does not produce infectious particles. The transduced cells were maintained in culture for 2 weeks to obtain stably integrated lines. Cells were then transfected with a plasmid expressing CRISPR/Cas9 and a puromycin resistance gene and HIV-1-specific (T5) or scramble (SC) guide RNAs (gRNAs). The T5 gRNA targets a highly conserved region between the TATA box and the NF-κB binding site in LTRs. CRISPR/Cas9-transfected cells were selected using high-dose puromycin. As shown in Fig. 1a, Luc activity dropped abruptly to less than 25% between days 2 and 3 after transfection and declined to nearly undetectable levels thereafter in cells treated with CRISPR/Cas9 and T5 gRNA. To rule out that Luc reduction was caused by cell death or an arrest of cell growth because of transfection, we performed a cell viability assay (WST-8) at 48 h posttransfection. As shown in Fig. 1b, no differences were observed among cell populations, thus demonstrating that Luc reduction was indeed due to HIV-1 editing.

To better understand how CRISPR/Cas9 editing affected HIV-1 transcription, we transduced 293T cells with VSV-G-pseudotyped NL4-3/GFP (green fluorescent protein) lentiviral particles, and 2 days later, we either left them as such (NL4-3/GFP) or transfected them with SC or T5 gRNA-containing CRISPR/Cas9. The Cas9-encoding plasmid contains red fluorescent protein (RFP) (mCherry) as a marker to label transfected cells (Fig. 1c). Consistent with the Luc data, the number of mCherry-positive (mCherry^+^)/GFP^+^ cells dropped to about 20% 4 days after transfection (Fig. 1d and e) as a consequence of CRISPR/Cas9 treatment. In addition, GFP was nearly undetectable by Western blotting at day 7 (Fig. 1f). No changes were observed after treatment with CRISPR/Cas9 combined with SC gRNA (Fig. 1a, b, and d to f).

### CRISPR/Cas9 LTR-targeted treatment increases circular HIV-1 genomes in transfected cells.

**FIG 2:**
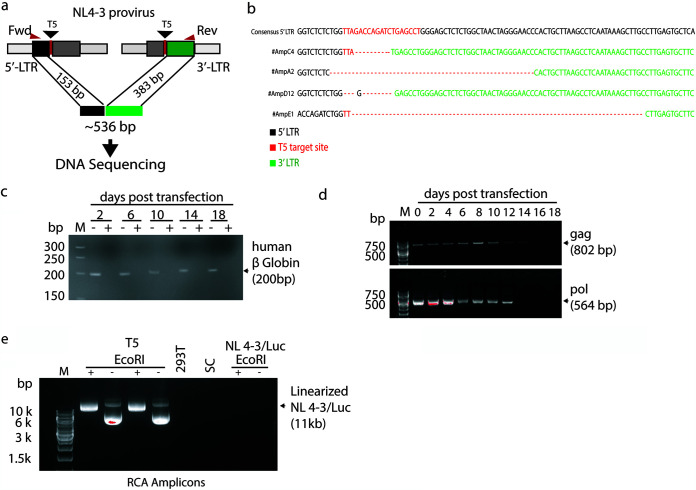
The excised HIV-1 provirus persists after circularizing and genomic DNA ends are repaired. (a) Localization of primers used to amplify the DNA fragments encompassing the CRISPR/Cas9 T5 cleavage sites. (b) Sequence data for four amplicons sequenced at the LTR junctions and compared to pNL4-3 by BLAST analysis. (c and d) PCR amplification of genomic DNA from NL4-3/Luc-transduced 293T cells for the β-globin gene (c) and *gag* and *pol* sequences of genomic DNA (d) after DNA exonuclease digestion every other day for 18 days. M, molecular weight marker. (e) Rolling-circle amplification (RCA) of DNA from NL4-3/Luc-transduced cells at day 10 after CRISPR/Cas9 and T5 or SC gRNA transfection. This technique allows the selective amplification of circularized DNA as concatemers, requiring digestion with a single cutter to obtain full-length fragments. RCA amplicons were electrophoresed as such (−) or after EcoRI digestion (+), which cuts NL4-3/Luc once in *pol*. 293T cells were used as a negative control.

To investigate what results from CRISPR editing, we first looked into how cellular DNA repair had resolved the excision events. We obtained individual clones of NL4-3/Luc cells by limiting dilution 5 days after HIV-1 excision. Seminested PCR amplification was performed with primers annealing upstream and downstream of the T5 target site, expecting an amplicon of 536 bp as a result of the mere joining of edited genomic DNA ends with no rearrangements in-between (Fig. 2a). As expected, PCR yielded amplicons of about 500 bp that were cloned and sequenced at random. As shown in Fig. 2b, most junctions were achieved by NHEJ and presented with deletions of DNA fragments of various lengths. Next, to investigate the fate of the HIV-1 genome after excision, genomic DNA was extracted from NL4-3/Luc-transduced 293T cells at various days after HIV-1 excision. To determine whether the excised proviral fragments circularized, genomic DNA was digested with DNA exonuclease to eliminate linearized DNA. As shown in Fig. 2c, no residual linear DNA could be found by checking for β-globin DNA amplification. Next, we PCR amplified *gag* and *pol*, and as determined by agarose gel electrophoresis, HIV-1 sequences were found in the exonuclease-digested DNA samples up to 14 days after HIV-1 excision (Fig. 2d). In contrast, no *gag* or *pol* sequences were found in the DNA samples from cells treated with SC gRNA and digested with the exonuclease (data not shown). These results suggest that upon excision, HIV-1 DNA persists for 14 days in circular DNA exonuclease-resistant forms. Since PCR analysis was performed in a population of dividing cells, the circular forms were likely to be less and less during subsequent cell mitoses; for this reason, it is not known whether the PCR signal waned because DNA molecules were eventually degraded or progressively diluted out.

To confirm this result, HIV-1 was excised from cells, and after 2 weeks, we examined the presence of circular DNA molecules by rolling-circle amplification (RCA) to enrich circular DNA molecules. RCA allows the selective amplification of circularized DNA as concatemers that can be digested with a single cutter to obtain single identical fragments. With this aim, NL4-3-transduced 293T cells were propagated for 10 days in the presence of puromycin to enrich for CRISPR-transfected cells and processed to extract whole DNA. Linear DNA was exonuclease-digested as described above and subjected to RCA to enrich for circular DNA molecules. Figure 2e shows the RCA amplicons electrophoresed as such or after digestion with EcoRI, which cuts NL4-3/Luc once. Upon transfection with T5 gRNA, 2 out of 5 CRISPR/Cas9-transfected samples contained RCA amplicons that, upon EcoRI digestion, yielded a discrete band. Of note, this band had a size compatible with that of the full-length NL4-3/Luc genome. The other 3 samples did not contain an LTR amplicon. Conversely, DNA from normal 293T cells or cells transduced with NL4-3/Luc, whether transfected or not with CRISPR/Cas9 plus SC gRNA, yielded no detectable circular DNA molecules (Fig. 2e). In all, these experiments indicate that circular HIV-1 proviral DNA appears after editing. They also demonstrate that the circularized HIV-1 genome persisted for a prolonged period of time without undergoing large deletions, as judged by the size of the EcoRI fragments.

### The excised HIV-1 genome generates concatemers through intermolecular joining.

**FIG 3:**
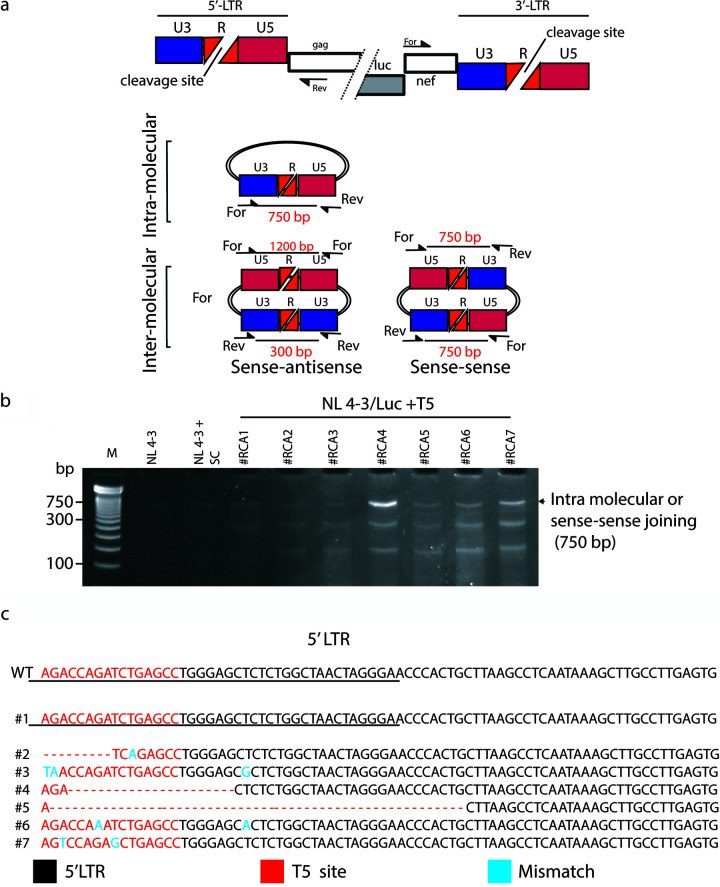
HIV intermolecular concatemers can be isolated on a large scale. (a) Schematic of circular molecules of HIV-1 provirus formed by a single molecule or two molecules bound together in the sense-sense or sense-antisense orientation and sizes of the amplicons of interest (in red), generated by the primers indicated. (b) PCR fragments obtained from RCA amplicons in 7 different experiments (#RCA1 to -7) using the primers shown in panel a. (c) Alignment of LTR sequences of the 750-bp fragments obtained from #RCA1 to -7 and retrieved from the agarose gel shown in panel b. The red sequences indicate the T5 gRNA annealing site. Dashes indicate base deletions, and blue letters denote nucleotide mismatches compared to the wild-type (WT) pNL4-3 sequence.

To evaluate whether the circularization of HIV-1 DNA had occurred intra- or intermolecularly, the EcoRI-digested RCA products (Fig. 2e) were extracted from the agarose gel and amplified in their LTR junctions. With this aim, we designed forward (Fwd) and reverse (Rev) primers annealing to the *luc*/*nef* and *gag* regions (Fig. 3a), respectively, and generating amplicons, the size of which depended on whether circularization had occurred within a single excised provirus (intramolecular) or between two or more molecules (intermolecular). In particular, as shown in Fig. 3a, a single, circularized molecule or two HIV-1 genomes bound in the sense-sense orientation would yield an amplicon of 750 bp. Conversely, two molecules bound in the sense-antisense orientation would yield two amplicons. The first, obtained by extending two Fwd primers, would be 300 bp, and the second, obtained with two Rev primers, would be 1,200 bp. Circular DNA generated by sense-sense intermolecular joining has the potential to recreate full-length LTRs, whereas a full 5′ LTR may be recreated by intramolecular end joining. Cells treated with T5 gRNA yielded more amplicons, two of which were compatible with a sense-sense circular DNA template (Fig. 3b). The 750-bp amplicons shown in Fig. 3b were then retrieved from the agarose gel and sequenced. Most of them had indels. Of note, one of them had a wild-type sequence, demonstrating that the intramolecular or intermolecular sense-sense orientation could recreate the original LTRs (Fig. 3c).

**FIG 4:**
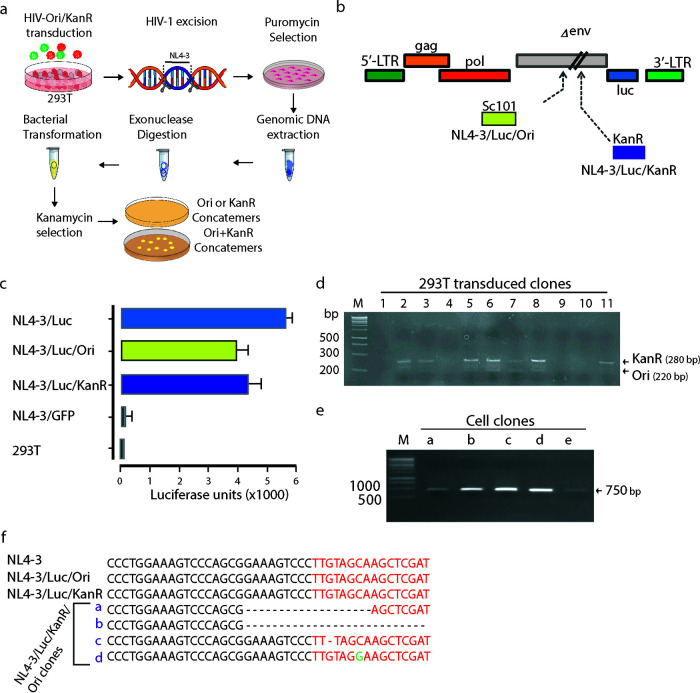
HIV-1 LTR circles can be extracted and amplified. (a) Schematic of the approach devised to select and identify intermolecular LTR HIV-1 concatemers. 293T cells were transduced with both HIV-Ori and HIV-KanR at an MOI of 5. A week later, their HIV-1 provirus was excised by CRISPR/Cas9 with T5 gRNA, and the edited cells were selected with puromycin. Next, their genomic DNA was extracted, digested by exonuclease to eliminate linear DNA, and used to transform bacteria. The recombinant bacteria were then selected on kanamycin-containing agar plates, where only intermolecular joining brought about growing colonies. (b) Graphic map of NL4-3/Luc/Ori and NL4-3/Luc/KanR as obtained by inserting the low-copy-number bacterial origin of replication SC101 (Ori) or the kanamycin resistance gene (KanR) into the pNL4-3/Luc backbone in *env*. (c) Evaluation of the transducing ability of lentiviral vectors as determined by luciferase activity in 293T cells transduced with VSV-G-pseudotyped NL4-3/Luc/Ori and NL4-3/Luc/KanR compared to NL4-3/Luc-transduced, NL4-3/GFP-transduced, and nontransduced 293T cells. (d) Screening of 11 cell clones doubly positive for NL4-3/Luc/Ori and NL4-3/Luc/KanR obtained by limiting dilution. For PCR, primers annealing to SC101 or KanR and pNL4-3 were used. (e) PCR amplification of the LTR junctions from NL4-3/Luc/KanR/Ori double-positive cell clones a to e. (f) Sequence analysis of clones a to e amplicons obtained in panel e. The red sequences indicate the T5 gRNA annealing site. Dashes indicate base deletions, and green letters denote nucleotide mismatches compared to the wild-type pNL4-3 LTR sequence.

To further confirm that circularization of excised HIV-1 genomes can also occur through the binding of two molecules in the sense-sense orientation, we devised the approach shown in Fig. 4a. Using the NL4-3/Luc backbone to prepare VSV-G-pseudotyped viral vectors, we constructed two different HIV-1-based vectors, named NL4-3/Luc/Ori and NL4-3/Luc/KanR (Fig. 4b). NL4-3/Luc/Ori contained the low-copy-number bacterial origin of replication SC101, and NL4-3/Luc/KanR carried the kanamycin resistance gene. The genes were cloned in the same restriction site within the Δ*env* strain (Fig. 4b). This would allow the identification of intermolecular concatemers generated after CRISPR/Cas9 excision because only intermolecular concatemers between the two constructs (Ori and KanR) could be used as plasmids to transform bacteria that would grow on kanamycin-containing agar plates. The respective VSV-G-pseudotyped particles (NL4-3/Luc/Ori and NL4-3/Luc/KanR) were functional, although they had a slightly reduced transduction capacity compared to NL4-3/Luc, as shown in Fig. 4c. Next, 293T cells were cotransduced with NL4-3/Luc/Ori and NL4-3/Luc/KanR and cultivated for 2 weeks to obtain stable transduction. Cells were cloned by limiting dilution and probed to select double-positive clones. Screening was performed with an upstream primer annealing to pNL4-3 and downstream primers annealing to Ori or KanR. Ori and KanR primers were designed to yield amplicons of about 220 and 280 bp, respectively, to discriminate clones by agarose gel electrophoresis (Fig. 4d). Cell clones scoring positive for both NL4-3/Luc/Ori and NL4-3/Luc/KanR were then treated with CRISPR/Cas9 and T5 gRNA, or SG gRNA, to excise both the NL4-3/Luc/Ori and NL4-3/Luc/KanR genomes. Clones were cultivated for 2 weeks in the presence of puromycin to select for CRISPR/Cas9 transfectants. Whole cellular DNA was then extracted and treated with exonuclease to eliminate linear DNA. To check if the excised NL4-3/Luc/Ori and NL4-3/Luc/KanR had formed intermolecular concatemers, which were named NL4-3/Luc/KanR/Ori, we took advantage of the KanR gene and SC101 to select and expand intermolecular concatemers in bacterial cells. Exonuclease-digested cellular DNA was thus used to transform bacterial cells, which were grown in the presence of kanamycin (Fig. 4a). Every transfection yielded ∼10 to 15 colonies; in this representative experiment, 5 random colonies, named clones a to e, were PCR checked for sense-sense intermolecular concatemers (Fig. 3b), as shown in the agarose gel in Fig. 4e. Next, they were sequenced at their LTR junctions (Fig. 4f). Sequence analysis confirmed that clones a to d were derived from excised NL4-3/Luc/Ori and NL4-3/Luc/KanR, which were bound in the sense-sense orientation. Clones a and b contained large deletions, and clones c and d had a single nucleotide deletion and a single mutation compared to the parental NL4-3 sequence, respectively. Parallel analyses performed with CRISPR/Cas9 and SC gRNA did not yield bacterial colonies.

In all, these results demonstrate that NL4-3/Luc/KanR/Ori may form upon the excision of NL4-3/Luc/Ori and NL4-3/Luc/KanR proviruses and that intermolecular, sense-sense joining may rebuild full-length LTRs.

### HIV-1 concatemers show perceptible transcriptional activity.

**FIG 5:**
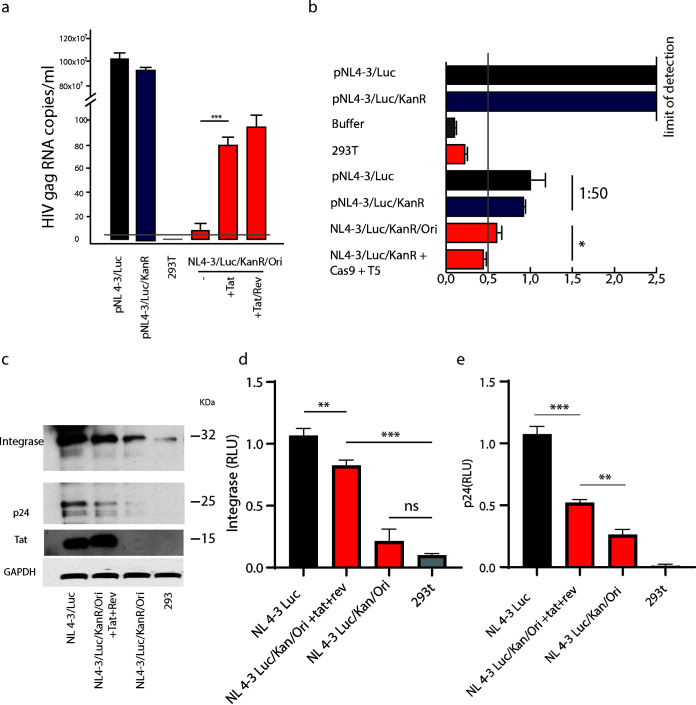
HIV-1 LTR concatemers exhibit detectable transcription in the presence of Tat and Rev. Analysis of HIV mRNA and protein production by NL4-3/Luc/KanR/Ori transfected alone or with Tat and Rev provided in *trans* was performed. (a) qRT-PCR quantitation of *gag* mRNA expression by cells transfected with pNL4-3/Luc, pNL4-3/Luc/KanR, or pNL4-3/Luc/KanR/Ori alone or after transfection of Tat alone or Tat plus Rev. Cells were lysed 3 days after transfection. The increment in *gag* mRNA expression after Tat or Tat plus Rev were provided in *trans* reached statistical significance (*P *= 0.0013). (b) Determination of p24 content by ELISA in lysates of cells transfected with pNL4-3/Luc or pNL4-3/Luc/KanR, as such or after a 1:50 dilution, or with NL4-3/Luc/KanR/Ori before and after CRISPR/Cas9 and T5 transfection. Isolated concatemers exhibited reduced but significant transcriptional activity. (c) Western blot analysis performed on lysates of cells transfected with pNL4-3/Luc or NL4-3/Luc/KanR/Ori, either with Tat plus Rev or alone, or nontransfected 293T cells. (d and e) statistical analyses of integrase (d) and p24 (e) expression in the Western blot shown in panel c. Data are shown as means ± SD and were analyzed by Student’s *t* test (*, *P* < 0.05; **, *P* < 0.01; ***, *P* < 0.001). RLU, relative luciferase units.

To understand whether the full-length LTRs in concatemers possess functional activity, 293T cells were transfected with either NL4-3/Luc/KanR/Ori (a pool of 3 HIV-1 circles with restored LTRs), pNL4-3/Luc, or pNL4-3/Luc/KanR. We then transfected the same cells with plasmids encoding Tat and Rev separately, with the aim of highlighting the transcription potentiality of HIV-1 circles by allowing export from the nucleus and translation. Cells were cultured for 5 days and then analyzed for HIV-1 *gag* mRNA expression by quantitative real-time PCR (qRT-PCR). NL4-3/Luc/KanR/Ori mRNA was detectable although at much lower levels than pNL4-3/Luc and pNL4-3/Luc/KanR (Fig. 5a). When HIV-1 p24 protein was measured in cell lysates, pNL4-3/Luc/KanR and pNL4-3/Luc cell lysates contained p24 levels that exceeded the highest limit of quantification of the enzyme-linked immunosorbent assay (ELISA) used. These samples were therefore diluted 1:50 for analysis. Interestingly, the NL4-3/Luc/KanR/Ori cell lysate p24 protein content was above the assay cutoff and in the range of those of pNL4-3/Luc/KanR and pNL4-3/Luc samples diluted 1:50 (Fig. 5b).

Because the p24 content was at the limit of detection, we hypothesized that this might be due to poor HIV-1 RNA transport from the nucleus to the cytoplasm. We do not know why *gag* RNA was barely detectable whereas p24 protein was seen (although again barely). We hypothesize that *gag* RNA could be stable enough to be translated over and over. Given that Tat and Rev exert the function of increasing the transcription and transport of HIV-1 transcripts to the cytoplasm (17, 18), we overexpressed Tat and Rev to see if the transcription of HIV-1 LTR circles would be highlighted. Western blot analysis of cell lysates, performed with an anti-HIV-1 polyclonal serum or anti-Tat antibody, showed weak production of Gag, Tat, and IN, confirming the transcriptional activity, although minimal, of NL4-3/Luc/KanR/Ori (Fig. 5c to e). Western blot analysis demonstrated Tat expression by pNL4-3/Luc, whereas it was almost undetectable in NL4-3/Luc/KanR/Ori-transfected cells. When NL4-3/Luc/KanR/Ori was cotransfected with pTat and pRev, the translation of concatemers was enhanced (Fig. 5d and e).

### Tat and Rev enhance HIV-1 expression of HIV-1 circular DNAs.

**FIG 6:**
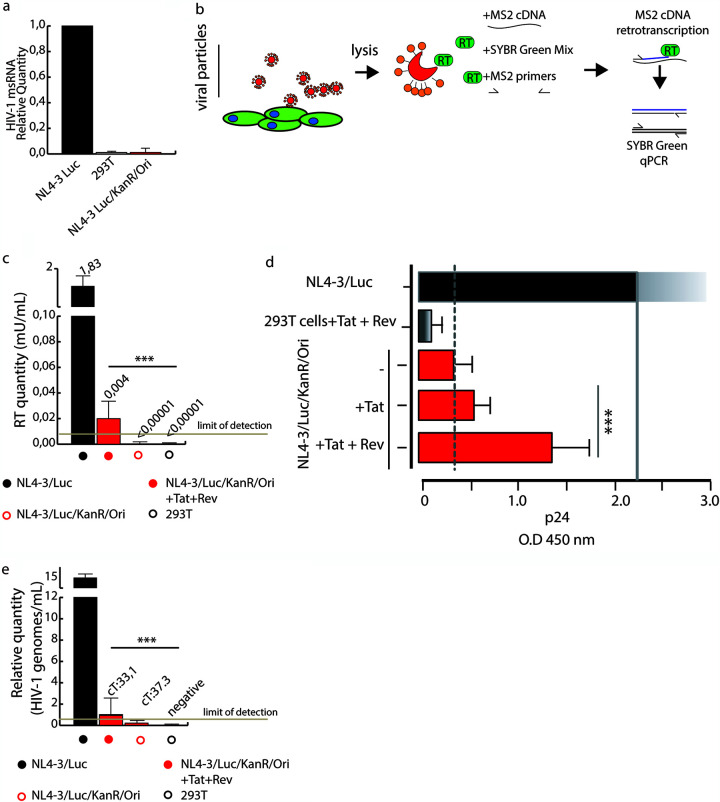
Infectious virions are produced from HIV-1 LTR concatemers once Tat and Rev are provided in *trans*. (a) TILDA, a method that detects multiply spliced RNA (msRNA), did not detect the production of msRNA by NL4-3/Luc/KanR/Ori-transfected cells. (b) Schematic representation of SG-PERT, which measures RT activity in supernatants. qPCR, quantitative PCR. (c) SG-PERT results of RT activity in the supernatants of cells transfected with NL4-3/Luc/KanR/Ori, pNL4-3/Luc, or NL4-3/Luc/KanR/Ori with or without Tat plus Rev. (d) Determination of the p24 content in the supernatants of cells transfected with NL4-3/Luc/KanR/Ori, pNL4-3/Luc, or NL4-3/Luc/KanR/Ori with or without Tat plus Rev. Data were collected from three independent experiments with four replicates each and analyzed by Student’s *t* test (***, *P *< 0.001). (e) Quantification of HIV-1 genomes in 293T cells transduced with the pseudotyped viruses analyzed in panels c and d (cT, threshold cycle). Statistical analysis was performed using Student’s *t* test. Data are expressed as means ± SD (*, *P* < 0.05; **, *P* < 0.01; ***, *P* < 0.001).

To shed light on the role of Tat and Rev in our model, we investigated whether the expression of unspliced HIV-1 mRNA was accompanied by the expression of multiply spliced HIV-1 mRNA (msRNA). We probed cell lysates for *tat*/*rev* msRNA, a marker that has been shown to reflect the ability of a cell to produce virus (19, 20). With this aim, we used the *tat*/*rev*-induced limiting dilution assay (TILDA), a method that discerns latently and productively infected CD4^+^ T lymphocytes (21). The TILDA measures the frequency of cells producing viral msRNA transcripts, either with or without a mitogenic stimulus, using serial dilutions of input cells. Based on the fact that *tat*/*rev* transcripts are generated after splicing full-length viral mRNAs, the TILDA reduces the likelihood of measuring proviruses with large internal deletions. As shown in Fig. 6a, NL4-3/Luc/KanR/Ori did not express *tat*/*rev* msRNA, in contrast to pNL4-3/Luc. This result, together with the low p24 intracellular content, suggested negligible viral replication in cells transfected with HIV-1 LTR concatemers only.

Next, we tested whether HIV-1 expression could be detected in terms of viral protein or particles. We cotransfected cells with NL4-3/Luc/KanR/Ori or pNL4-3/Luc and pVSV-G to obtain VSV-G-pseudotyped lentiviral vectors. At day 2 posttransfection, the supernatants were collected and assayed for the presence of reverse transcriptase (RT) activity (Fig. 6b and c), p24 (Fig. 6d), and pseudotyped particles (Fig. 6e). RT activity, tested using a SYBR green PCR-enhanced RT (SG-PERT) assay (22), was negative for HIV-1 LTR concatemers alone (Fig. 6c). The overexpression of Tat and Rev restored RT activity in the supernatants, even if at low levels. In agreement, p24, assayed by an ELISA, yielded an absorbance value slightly above the cutoff (Fig. 6d). The production of infectious particles was examined by using supernatants to transduce 293T cells that were then probed for the presence of NL4-3 provirus at day 3 posttransduction. This was assessed using Xpert HIV-1 Qual, a highly sensitive qualitative real-time PCR assay approved for *in vitro* diagnostics. In contrast to cells transfected with pNL4-3/Luc or NL4-3/Luc/KanR/Ori alone, which scored positive and negative, respectively, cells transfected with NL4-3/Luc/KanR/Ori+Tat+Rev yielded an amplification signal about 3 logs lower than that of the positive control, suggesting that about 1 out of 1,000 cells were transduced (Fig. 6e). These results confirm that intermolecular concatemers are unable to produce infectious virions *per se* but that the provision of Tat and Rev may restore infectivity although at a minimal level.

### HIV-1 provirus can be excised from latently infected J-Lat cells by CRISPR/Cas9 editing.

**FIG 7:**
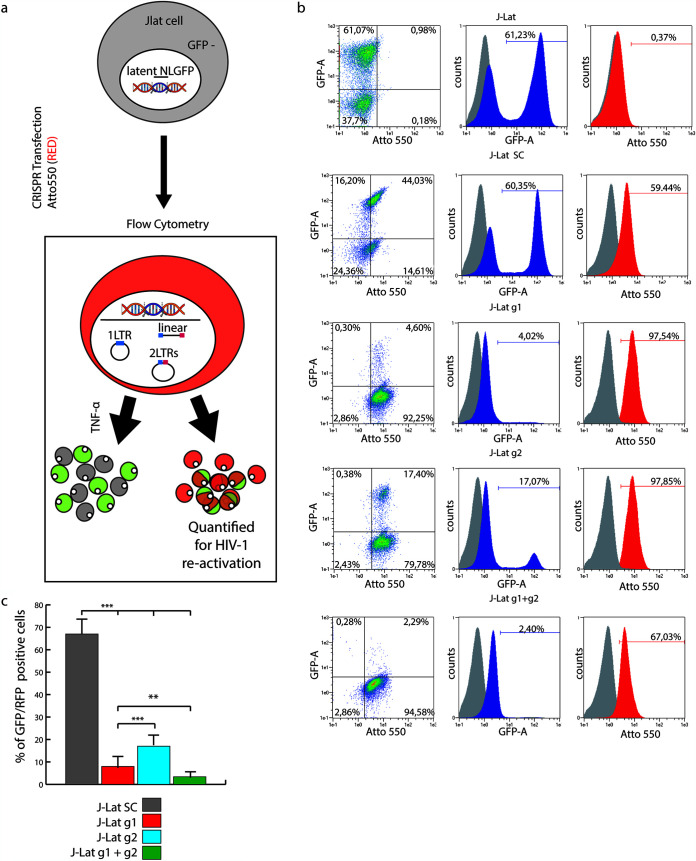
HIV-1 provirus is also excised in latently infected J-Lat T cell leukemia cells by CRISPR/Cas9 editing. (a) Schematic illustration of J-Lat treatment. J-Lat cells were first transfected with g1, g2, g1+g2, or SC gRNA complexed with fluorescently labeled tracr-RNA (Atto550) and Cas9. Twenty-four hours later, cells were exposed to TNF-α to activate HIV expression. Next, J-Lat cells were analyzed by FACS analysis. (b) Flow cytometry analysis of J-Lat cells left untransfected or transfected with SC, g1, g2, or g1+g2. All cells were activated with TNF-α 24 h after transfection. Ungated events are analyzed (dot plots on the left). The gray histogram in the overlays shows untreated, latent J-Lat cells. (c) Histogram plot of the data in panel b. The number of GFP^+^ cells is significantly lower upon RNP treatment with g1, g2, and g1+g2 than with SC gRNA. Combined treatment with g1+g2 or treatment with g2 alone is the most or the least effective, respectively, in reducing activated GFP^+^ cells. Statistical analysis was performed by one-way ANOVA, and data are expressed as means ± SD (***, *P* < 0.001); multiple comparisons were obtained by Bonferroni’s *post hoc* test.

HIV-1 excision by CRISPR/Cas9 was conceived to cure latently infected cells, and most studies target these cells (3, 4, 7, 16). With the aim of monitoring the fate of the excised provirus in an *in vitro* model that closely mimics natural HIV-1 infection, we repeated some experiments using J-Lat cell clone 9.2, a human T cell leukemia line derived from Jurkat, harboring a single copy of HIV-R7/E^−^/GFP, a full-length integrated HIV-1 genome expressing GFP (23), per cell. In such cells, HIV-R7/E^−^/GFP persists in a latent phase, which can be reverted by treating cells with tumor necrosis factor alpha (TNF-α) or exogenous Tat (24). Due to the low sensitivity of T cells to lipofection (25, 26), J-Lat cells were CRISPR/Cas9 transfected using the ribonucleoprotein (RNP) nucleoporation system. For this purpose, we adapted the previously used gRNAs to the RNP transfection method. The first guide, named g1, targets the U5 region of the LTR, and the second, g2, binds the same R site targeted by T5 gRNA. As a negative control, we used a nonrelated gRNA (SC). J-Lat cells were transfected with RNP combined with SC, g1, g2, or g1 plus g2 (g1+g2) labeled with Atto550. This dye allows the tracking of RNP-transfected cells, which appear fluorescently labeled in red. Twenty-four hours after RNP treatment, HIV-1 GFP expression was induced by TNF-α, as shown in Fig. 7a. HIV-1 reactivation was quantified in edited cells by enumerating GFP^+^ Atto550^+^ cells by flow cytometry, performed 24 h after RNP treatment (Fig. 7a). Nontransfected, nonactivated J-Lat cells had no red fluorescence and traces of GFP fluorescence (<1%) (Fig. 7b, gray overlay histograms). TNF-α induction caused GFP^+^ cells to increase to 61.2% (Fig. 7b, top dot plot). SC gRNA treatment did not affect HIV activation, as cells were roughly 60% GFP^+^ (Fig. 7c). Treatment with g1 or g2 reduced HIV-1 activation by TNF-α to roughly 8% (Fig. 7c, red bar) or 20% (Fig. 7c, blue bar) GFP^+^ cells, respectively. We show that multiple targeting of LTRs has a stronger effect in reducing HIV-1 activation than single targeting since g1+g2 proved to reduce GFP^+^ cells to roughly 5% (Fig. 7c, green bar). Thus, T cell leukemia cells can also be efficiently edited by the appropriate gRNAs.

### HIV-1 LTR circles accumulate in a latently infected T cell leukemia line after excision.

**FIG 8:**
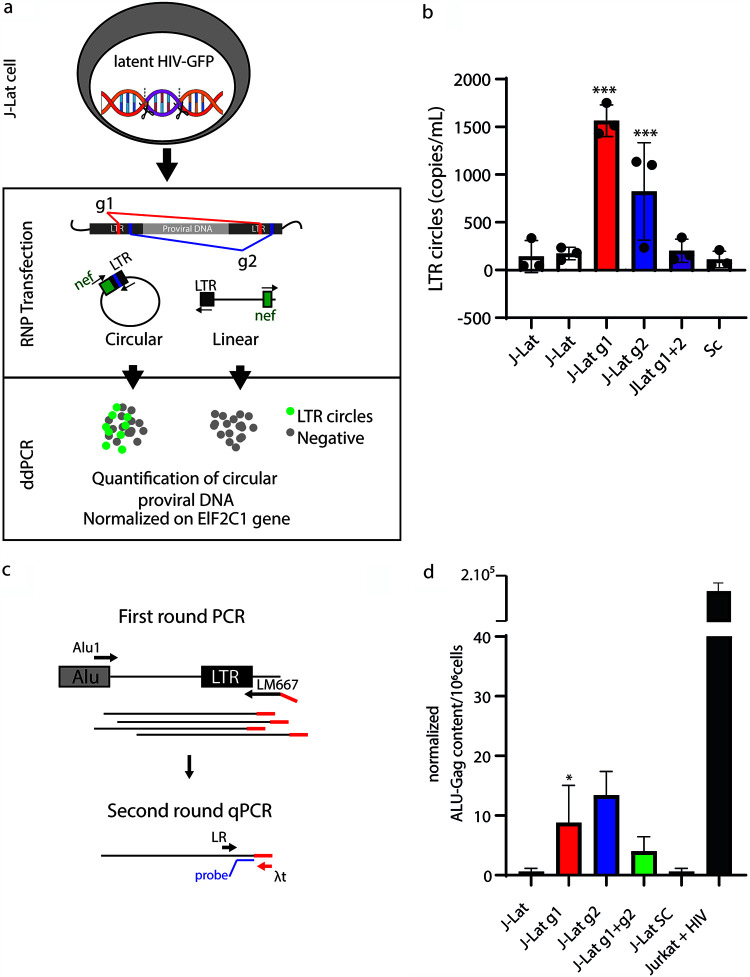
HIV-1 LTR circles are also formed in HIV-1 latently infected J-Lat T cell leukemia cells. (a) Schematic of the experimental procedure. Briefly, ddPCR was performed on genomic DNA extracted 24 h after CRISPR/Cas9 editing. DNA was digested with BseJI using primers for HIV *nef* and LTRs, which allow the amplification of circles only. (b) Histogram plot of the average number of LTR circle molecules from 3 independent experiments (black circles), performed as described above for panel a. g1 and g2 gRNAs (red and blue histograms, respectively) led to substantial increases in LTR circles, whereas double-LTR targeting (purple histogram) did not. (c) PCR was performed on genomic DNA extracted from g1+g2-edited J-Lat cells as described above for panel a. Briefly, *Alu*-*gag* preamplification was performed to add the λt tag, and a second round amplified LTR-λt to avoid amplifying nonintegrated proviral DNA. (d) Histogram plot of *Alu*-*gag* content in untreated and g1-, g2-, g1+g2-, and SC-treated J-Lat cells. Statistical analysis of ddPCR values was performed using one-way ANOVA, and results are expressed as means ± SD (*n* = 3) (*, *P* < 0.1; ***, *P* < 0.001).

To monitor the formation of LTR circular molecules, we used droplet digital PCR (ddPCR) as described previously (27). The primers and probe were designed in such a way as to detect *nef*-LTR junctions, i.e., LTR circle molecules (depicted as green dots in Fig. 8a), as opposed to linear excised molecules that were not amplified (gray dots). Results were obtained from cellular DNA extracted 24 h after CRISPR/Cas9 treatment and normalized using primers and a probe targeting the housekeeping EIF2C1 gene (Fig. 8a).

J-Lat cells were first transfected with RNPs combined with g1, g2, g1+g2, and SC guide RNAs. Next, total DNA was extracted 24 h later. Figure 8b shows that LTR circles increase by 7-fold after CRISPR/Cas9 cleavage with g1 and, to a lesser extent, g2 (2- to 3-fold increase). Interestingly, double targeting of LTRs almost abolished HIV-1 LTR circle amplification, possibly due to primer binding site disruption (Fig. 8b). These results show that not all gRNAs are equal. These experiments indicate that circularization of the excised provirus also occurs in cells harboring one integrated HIV-1 genome. Due to the biology of HIV-1, it is unlikely that integrase is involved in the circularization and reintegration of these excised elements. Despite that, in the context of genomic instability due to unpredictable off-target activity, we hypothesize that cellular proteins involved in the NHEJ pathway and HDR might allow reintegration events of excised provirus. To probe possible reintegrations in the genome of J-Lat cells, in the context of latent infection, we took advantage of *Alu* PCR. *Alu*-LTR amplification was chosen so as to not amplify the single provirus integrated into J-Lat 9.2 cells, which has been mapped far from *Alu* sequences, i.e., within the PP5 gene, chromosome 19, by two independent studies (28, 29). *Alu* PCR therefore would not amplify the original provirus but rather would amplify those that integrated into a new site close to an *Alu* region in the genome (Fig. 8c). Figure 8d shows that, although at a minimal frequency, reintegration occurs after g1 and g2 editing.

### LTR circles are also increased by LTR targeting in acutely infected cells.

**FIG 9:**
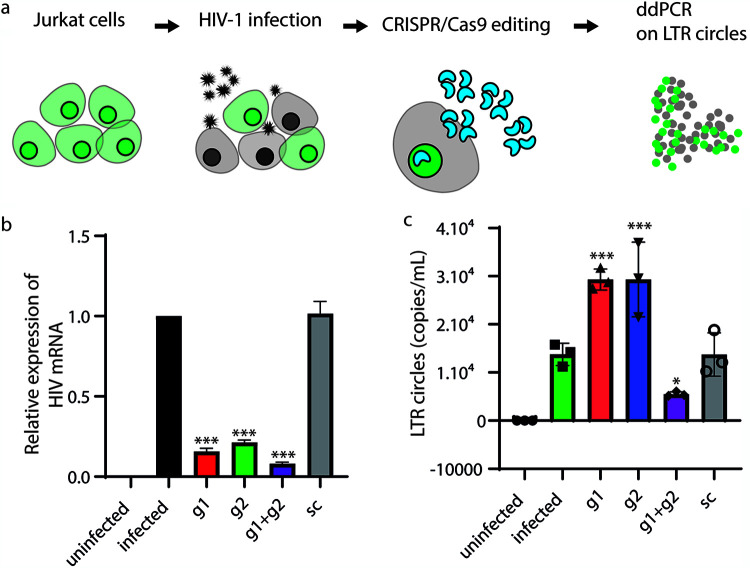
The provirus of a clinical HIV-1 isolate is excised and circularizes after CRISPR/Cas9 editing. (a) Schematic illustration of the experimental procedure. Jurkat cells were infected with an HIV-1 clinical lymphotropic isolate. Twenty-four hours after infection, cells were electroporated with Cas9 g1, g2, g1+g2, or SC RNP and processed for ddPCR 24 h after transfection. (b) RT-PCR of viral RNA extracted from cell lysates described above for panel a 12 days from CRISPR/Cas9 transfection. The provision of both g1 and g2 RNPs decreased HIV-1production. Again, LTR double targeting increased the efficiency of HIV-1 eradication. The *y* axis shows copies of HIV-1 genomes per milliliter of the supernatant. Statistical analysis was performed using one-way ANOVA with a *post hoc* Tukey test; data from experiments are expressed as means ± SD (*, *P *< 0.1; **, *P *< 0.01; ***, *P *< 0.001). (c) ddPCR shows an increase in LTR circles in Jurkat cells transfected with g1 or g2 RNPs. The provision of both g1 and g2 RNPs decreased the amount of LTR circles after editing. The *y* axis shows the LTR circle concentration expressed as LTR circle copies per milliliter.

To confirm our findings in acutely infected T cell leukemia cells, we repeated the editing using Jurkat cells that had been infected with a clinical lymphotropic strain of HIV-1 at a multiplicity of infection (MOI) of 0.05. As for J-Lat cells, we used ddPCR to detect LTR circles 24 h after CRISPR/Cas9 nucleoporation. As shown in Fig. 9b, all the guides targeting LTRs dramatically reduced the number of HIV-1 transcripts detected in cell lysates 24 h after transfection, confirming the efficacy of CRISPR/Cas9 in curbing HIV-1 progression. Interestingly, we observed an increase in LTR circles when HIV-1 proviral DNA was excised with single gRNAs. Integration of excised provirus might also occur in this context, but it cannot be distinguished easily from the originally integrated HIV-1 genome.

## DISCUSSION

CRISPR technology is becoming the leading gene-editing tool, with increasingly expanding fields of application (30, 31). These include HIV therapy, where excision of the integrated HIV-1 genome from cellular DNA can be achieved by taking advantage of CRISPR/Cas9 site-specific cleavage. The ends of cellular DNA are then joined by NHEJ (4). This approach has proven to be effective and potent *in vitro*, whereas a number of limitations may be relevant when it is transposed *in vivo* (3, 4). CRISPR/Cas9 may lead to possible off-target activity and gene rearrangement following DNA repair (32, 33); moreover, diversity and mutations of the HIV-1 genome may constrain target selection (7). Finally, the site of integration and transcriptional activity of the provirus may impact susceptibility to CRISPR cleavage (34). *In vivo*, this scenario is further complicated by the limited availability of effective delivery vehicles (35). One of the difficulties of CRISPR strategies is the control of the type of editing in cells and of the ensuing NHEJ process. Targeting the HIV-1 LTR alone has been repeatedly shown to rapidly lead to viral escape (8, 36). Thus, to effectively eliminate HIV-1 infection *in vivo*, it would be advisable to convey multiple RNA guides into a single cell and ensure chopping of the provirus. Although rapid progress in CRISPR/Cas9 technology and improvement of delivery strategies will provide a way to circumvent these drawbacks, the use of multiple guides itself increases off-target activity with unpredictable DNA rearrangements.

The main aim of this study was to monitor the fate of the HIV-1 provirus once it is excised and the cellular repair mechanisms that are triggered to heal the scars generated by CRISPR/Cas9. For this purpose, the experimental design was structured in two main parts. First, the fate of HIV-1 provirus was studied in 293T cells transduced with a replication-defective HIV-1-based vector. Second, the findings were confirmed in a human T cell leukemia line that contains one copy of integrated HIV-1 per cell and is a well-established model to study latency and reactivation (37, 38). Finally, further confirmation was achieved in T cell leukemia cells infected with a clinical lymphotropic isolate of HIV-1. We found that, if cut with a single gRNA, the excised HIV-1 provirus persisted in cells for a protracted period. The excised linear provirus differed from the linear HIV-1 cDNA produced by RT during viral replication *in vitro* and *in vivo* because it missed portions of the LTRs at both ends. However, circularized HIV-1 proviral DNA could also be found, originating from either single molecules or the concatemerization of HIV-1 cDNAs that persisted as episomes in infected cells where they dimerized. In this case, they reconstituted functional LTRs at both ends. When a single proviral genome is excised, only one LTR is formed.

Similar to our observations after CRISPR/Cas9 proviral excision, HIV-1 episomes have been found *in vitro* (39–41) and in patients at advanced phases of the disease (14). They are believed to be the result of abortive integration processes (42). In keeping, episomal HIV-1, which can also be found during antiretroviral regimens, has been postulated as a marker of ongoing *de novo* infection that may trigger viral rebound after treatment interruption (43). Unintegrated HIV-1 is abundant in resting, nonproliferating CD4^+^ T cells and yields *de novo* virus production following cytokine exposure of resting cells (44). Interestingly, episomal HIV-1 DNA was found to express early gene (*rev*-independent) msRNAs at a low level, and upon superinfection, it could also express late (*rev*-dependent) genes, indicating that it has the full potential for transcription (45). For this reason, the unintegrated DNA may influence viral RNA decay consequent to therapy and even lead to recombination with a second incoming virus, thus contributing to the generation of viral diversity (46).

The results of our study support the observation that CRISPR/Cas9 gene editing is an extremely powerful technique to excise HIV-1. However, our work demonstrates that CRISPR/Cas9-excised provirus persists in cells for at least 2 weeks after editing. In transduced 293T cells, we found that two or more linear fragments could bind together in a sense-sense orientation and express unspliced *gag* mRNA at low levels. Importantly, this viral DNA responded to exogenous Tat and Rev, as described previously for nonintegrated HIV-1 DNA (47). Tat alone remarkably increased the level of expression of unspliced mRNA, but when tested for protein levels, Rev made the real difference, as it significantly augmented the amount of p24 protein. Furthermore, when provided with VSV-G as an envelope protein, together with Tat and Rev, LTR circles can produce infectious VSV-G-pseudotyped virions. Although in these experiments, the production of transducing particles, mimicking infectious virions, was at the limit of detection, this may strengthen the caveat posed by the persistence of proviral DNA after CRISPR/Cas9 editing in the absence of antiretroviral therapy. It might be argued that Tat and Rev, transfected into cells to facilitate the measurement of concatemer products, led to much higher protein levels than with superinfection. Moreover, superinfection may be considered a rare event *in vivo*, but how rare it really is remains poorly defined. Besides superinfection by a second strain, which is indeed rare, Gelderblom and colleagues showed that the same HIV-1 strain can reinfect the cell, a process known as coinfection (47).

Concatemer formation reconstituting functional LTRs was detected after CRISPR/Cas9 in 293T cells. This might be due to the fact that multiple copies of provirus are present, a condition that is not so rare and has been observed *in vivo* (48). For example, when HIV-1 infection spreads through virological synapses, i.e., adhesive structures between infected and uninfected cells, multiple copies of HIV-1 are transmitted to the engaged uninfected cells (49, 50). This phenomenon occurs in experimental models and in patients (51, 52) and has been linked to reduced sensitivity to antiretrovirals (49).

To understand what happens in lymphoid cells, we repeated the experiments in (i) J-Lat cells, an HIV-1-infected T cell leukemia line harboring one HIV-1 copy per cell, which persists in a latent state, and (ii) Jurkat cells infected with a clinical lymphotropic isolate of HIV-1. These cells are derived from a T cell leukemia line and are established models to study HIV infection in T cells (28, 29). Repeating experiments on primary T cells would have been very informative; unfortunately, we had to resort to T cell leukemia lines because infection/transfection of human concanavalin A (ConA)-stimulated peripheral blood mononuclear cells did not give us sufficient events for further analyses to have a significant number of cells. As in 293T cells, excision through HIV-1 LTRs determined a sharp and statistically significant increment in episome numbers. In J-Lat cells, we found the same persistence of single LTR circles. Even if CRISPR/Cas9 efficiently eradicated infection in the large majority of J-Lat cells, we observed novel integrations of excised provirus in the proximity of *Alu* sites. This phenomenon can be explained by dissecting the biology of HIV-1 replication: even in the unlikely possibility that latent provirus might reactivate transcription in the absence of TNF-α, it is even more unlikely that the newly encoded Gag-Pol protein might be active without HIV-1 protease cleavage. The most plausible reason to explain the reintegrations that we observed is the genomic instability of CRISPR-transfected cells. Even if this was a rare event, it is actually possible that some off-target cuts share partial homology with excised DNA elements. In this context, HDR, a mechanism that recombines DNA elements when the double-strand DNA is cleaved, might facilitate reintegration. This is why we suggest targeting HIV-1 DNA in several distinct sites, even if it is unlikely that these “crippled” LTR elements can be active again.

Interestingly, circles were indeed also present in Jurkat cells after infection with a clinical HIV-1 strain, no matter whether the virus was productively replicating or dormant. These data show that circles form without activation, even if at an obviously much lower number. These data suggest that cutting DNA, thereby activating NHEJ, brings about the formation of LTR circles.

In conclusion, we provide evidence that if the HIV-1 genome is excised as a single fragment, it persists and reorganizes in concatemers. Most concatemers and episomes are likely to be lost during subsequent cell mitoses and have limited persistence in dividing cells but are nonetheless a warning note. This work stresses the importance of the CRISPR/Cas9 strategy in the cure of HIV-1 and should be a stimulus to (i) implement the efficacy of delivery systems and CRISPR/Cas9 strategies *in vivo* to achieve cleavage of the HIV-1 genome at multiple sites and in all cells, no matter where the provirus is integrated and how many copies are present within the cell, and (ii) prolong antiretroviral treatment to avoid the expression of excised proviral forms.

## MATERIALS AND METHODS

### Cell cultures and plasmids.

The human 293T cell line was purchased from the American Type Culture Collection (Manassas, VA) and cultured in Dulbecco’s modified Eagle’s medium (DMEM) supplemented with 10% fetal bovine serum (FBS), 2 mM l-glutamine, and antibiotics (penicillin and streptomycin) at 37°C with 5% CO_2_. The human T cell leukemia J-Lat cell line was obtained through the NIH AIDS Reagent Program, Division of AIDS, NIAID. J-Lat cells were produced by transducing Jurkat cells with HIV-R7/E^−^/GFP at a low MOI in such a way to generate clones containing one copy of integrated HIV per cell. Cells were cultured in RPMI 1640 supplemented with 10% FBS, 2 mM l-glutamine, and antibiotics at 37°C with 5% CO_2_. J-Lat HIV-R7/E^−^/GFP, which is a full-length HIV-1 genome with a nonfunctional Env due to a frameshift and GFP in place of the Nef gene, generates incomplete virions. HIV-R7/E^−^/GFP is activated for transcription and expresses GFP by treatment of J-Lat cells with tetradecanoyl phorbol acetate (TPA), TNF-α, or exogenous Tat (24). Jurkat cells were grown in RPMI 1640 supplemented with 10% FBS, 2 mM l-glutamine, and antibiotics at 37°C with 5% CO_2_. pNL4-3/Luc (https://www.aidsreagent.org/pdfs/ds3418_010.pdf) (catalog number 3418) and pNL4-3/GFP (https://www.aidsreagent.org/11100_003.pdf) (catalog number 11100) were obtained through the AIDS Reagent Program. The first is an HIV-1 NL4-3 luciferase reporter vector that contains defective Nef, Env, and Vpr; it is competent for a single round of replication. It can produce infectious virus only after cotransfection with an *env* expression vector. The second is also derived from pNL4-3 but carries enhanced green fluorescent protein (EGFP) in the *env* open reading frame. This vector expresses an endoplasmic reticulum (ER)-retained truncated Env-EGFP fusion protein. NL4-3/Luc is derived from pNL4-3 Luc.R^−^E^−^ and encodes luciferase (Luc) (17). NL4-3/GFP is derived from pNL4-3 ΔEnv EGFP and expresses GFP as an Env-GFP fusion protein that is retained in the endoplasmic reticulum (17). pNL4-3/Luc/Ori and pNL4-3/Luc/Kan were produced by molecular cloning into pNL4-3/Luc. Both SC101, the low-copy-number bacterial origin of replication, and KanR were extracted from pSF_CMV-SC101 (catalog number OG13; Oxford Genetics, Oxford, UK) following digestion with SwaI (SC101) and PmeI (KanR). Fragments were cloned into the BseJI site of pNL4-3 *env*. Recovery-of-transcription experiments were performed using pTat (catalog number 164442; Addgene, MA) and pRev (catalog number 119322; Addgene, MA). All lines were tested for *Mycoplasma* contamination (59).

### Digestion of host and linear DNA.

Host and proviral DNAs were extracted from cells using a standard phenol-chloroform method. Briefly, we added 1 volume of phenol-chloroform-isoamyl alcohol (25:24:1) per sample, vortexed the mixture for 20 s, and then centrifuged the mixture for 5 min at 16,000 × *g*. The aqueous phase containing total DNA was purified by ethanol precipitation. Three micrograms of purified DNA was treated with 2 μl of Plasmid-Safe ATP-dependent exonuclease (Epicentre, Madison, WI, USA) at 37°C for 30 min and then heat inactivated by a 30-min incubation at 70°C.

### RCA.

Following Plasmid-Safe ATP-dependent exonuclease treatment, circular DNA molecules were amplified with random hexamers and TempliPhi DNA polymerase (Merck KGaA, Darmstadt, Germany) according to the manufacturer’s instructions. Briefly, the reaction mixture containing 10 ng DNA was incubated at 30°C for 6 h. The reaction was then blocked by heat inactivation at 65°C for 5 min.

### CRISPR/Cas9 design and transfection.

HIV-1 Cas9 guides were designed using the Benchling algorithm and ranked based on specificity and efficiency. The selected gRNA, named T5 (TTAGACCAGATCTGAGCCT), targets the LTR R region and is 99 to 100% conserved in all HIV clinical isolate sequences available in the Los Alamos database at the end of 2015 (data not shown). T5 gRNA was cloned into pSpCas9-2A-Puro (catalog number 62988; Addgene, MA, USA) or pU6-Cas9-T2A-mCherry (catalog number 64324; Addgene) according to a standard protocol (53). Transfection of DNA plasmids into 293T cells was performed with a standard calcium phosphate method. J-Lat cells were transfected with CRISPR/Cas9 RNP and *trans*-activating CRISPR RNA (tracr-RNA) Atto550 (IDT, Coralville, IA) by electroporation (Neon electroporation system; Thermo Fisher, MA, USA) using the following parameters: 1,400 V, width of 10 ms, and 3 pulses.

Guide 1 (g1) and g2 were designed using the IDT algorithm, and the sequences are as follows: /AlTR1/rUrGrArCrArUrCrGrArGrCrUrUrUrCrUrArCrArArGrUrUrUrUrArGrArGrCrUrArUrGrCrU/AlTR2/ for g1 and/AlTR1/rArCrUrCrArArGrGrCrArArGrCrUrUrUrArUrUrGrGrUrUrUrUrArGrArGrCrUrArUrGrCrU/AlTR2/ for g2. Both gRNAs target the LTR, g2 targets the same R site as T5, and g1 targets U5.

### 293T cell transduction.

Transduction of 293T cells was performed with VSV-G-pseudotyped particles. These were produced by transfecting 293T cells with pNL4-3 or its derivatives described above and a VSV-G plasmid. Third-generation lentiviral vectors were obtained by transfecting 293T cells with pREV (catalog number 119322; Addgene, MA), pGagPol (catalog number 164441; Addgene, MA), and pHIV-1eGFP (catalog number 21373; Addgene, MA). The generated particles were harvested at day 2 or 3 posttransfection and titrated as described previously (53). Transduction was performed at an MOI of 5 to transduce 2 × 10^5^ 293T cells. Cloning of transduced 293T cells was performed in 96-well plates at the indicated days posttransduction.

### HIV-1 infection.

Infection of Jurkat cells with a lymphotropic HIV-1 clinical strain was performed at an MOI of 0.05. The clinical strain was isolated on MT2 cells and showed a syncytium-inducing phenotype, CXCR4 tropism as determined by sequencing of the V3 *env* region, and a wild-type genotype of *pro* RT and *int* regions. This strain was isolated from an HIV-infected patient at the Laboratory of Virology, Department of Molecular Medicine, Sapienza University of Rome, Rome, Italy (54).

### ddPCR.

For ddPCR analysis, total DNA from 5 × 10^5^ J-Lat cells was purified using a Qiagen blood minikit (Qiagen, Hilden, Germany) and quantitated spectrophotometrically. Aliquots of 2.5 μg were then BseJI digested (Thermo Fisher, MA, USA) to fragment the genomic DNA without cutting the LTR junctions. Restriction products were column purified using a PCR cleanup kit (Qiagen), and 2.5 ng of purified DNA was amplified by ddPCR using the following primers and probe for LTR circles: Fwd primer AACTAGGGAACCCACTGCTTAAG, Rev primer TCCACAGATCAAGGATATCTTGT, and probe 6-carboxyfluorescein (FAM)-ACACTACTTGAAGCACTCAAGGC. The reaction was performed as follows: 10 min at 95°C for denaturation and 40 cycles of 95°C for 30 s and 60°C for 60 s, followed by 98°C for 10 min. After the completion of PCR cycling, reaction mixtures were placed in a QX200 instrument (Bio-Rad, Milan, Italy), and droplets were analyzed according to the manufacturer’s instructions. Normalization was performed by targeting the EIF2C1 gene using a premade probe solution (Bio-Rad) according to the manufacturer’s instructions.

### Concatemer isolation, characterization, and sequencing.

The selection of clones doubly positive for pNL4-3/Luc/Ori and pNL4-3/Luc/Kan was performed by PCR amplification using the following primers: Env Fwd (GACACAATCACACTCCCA), Kan Rev (AATAGCCTCTCCACCCAA), and Ori Rev (TGTGGTGCTATCTGACTT). Selected clones were then transfected with the T5 gRNApspCas9 plasmid (catalog number 459; Addgene) and cultivated in the presence of puromycin (3 μg/ml) to select for transfected cells. Following DNA extraction and digestion with ATP-dependent exonuclease, 50 to 200 ng of total DNA, quantitated before exonuclease digestion, was used to transform 10^8^ ultracompetent Stbl2 Escherichia coli cells (Invitrogen, Carlsbad, CA, USA). Transformants were seeded in Luria-Bertani (LB) agar plates supplemented with kanamycin (50 μg/ml). Bacterial colonies were picked and screened to eliminate spurious clones containing residual pNL4-3/Luc/Kan, which also harbors the ampicillin resistance gene in the plasmid vector. With this aim, colonies were split and seeded into two LB broth cultures containing kanamycin or ampicillin. Clones growing only in kanamycin medium were expanded, and concatemers were extracted and purified using Maxi prep (Qiagen). Recovered DNA was tested by PCR to confirm the LTR junction using the following primers: Fwd primer AACTAGGGAACCCACTGCTTAG and Rev primer GACAAGATATCCTTGATCTGTGGA. Positive clones were sequenced in LTR junctions by cycle sequencing.

### Biological activity of excised proviruses and concatemers.

Analyses were focused on the detection and quantitation of HIV mRNAs and proteins and performed on cell lysates obtained by transfecting 1 × 10^5^ 293T cells with 0.5 to 1.0 μg of pNL4-3 derivatives. The same analyses and infectivity as the released virions were assessed with 1 × 10^5^ 293T cells transfected with 0.5 to 1.0 μg pNL4-3 derivatives and 0.1 μg VSV-G plasmid. Total cellular RNA was extracted using a Maxwell 16 LEV simply RNA extractor (Promega, Madison, WI, USA). Total proteins were extracted using radioimmunoprecipitation assay (RIPA) buffer (0.22% beta-glycerophosphate, 10% Tergitol NP-40, 0.18% sodium orthovanadate, 5% sodium deoxycholate, 0.38% EGTA, 1% SDS, 6.1% Tris, 0.29% EDTA, 8.8% sodium chloride, 1.12% sodium pyrophosphate decahydrate).

### Measurement of HIV RNA and HIV DNA.

Intracellular and supernatant HIV RNAs were quantitated using Cobas AmpliPrep and Cobas-6800 (Roche, Milan, Italy), respectively. Both platforms and tests are routinely used at the Virology Unit, Pisa University Hospital; are certified for *in vitro* diagnostics; and detect up to 20 HIV RNA copies/ml. HIV msRNA was detected and quantitated using a TILDA (20). Briefly, total RNA extracted as described above was reverse transcribed at 50°C for 15 min, denatured at 95°C for 2 min, and amplified for 24 cycles (95°C for 15 s and 60°C for 4 min) on a T100 PCR instrument (Bio-Rad, Hercules, CA, USA). At the end of this process, samples were diluted to 50 μl with Tris-EDTA buffer, and 1 μl of the sample was used as the template for a second *tat*/*rev* real-time PCR. Primer sequences and details to calculate means and standard deviations of Δ*C_T_* (threshold cycle) values were described previously (21). Proviral DNA in 293T cells was assayed with Xpert HIV-1 Qual, manufactured by Cepheid (Milan, Italy) and certified for *in vitro* diagnostics. This assay has a sensitivity of 278 copies/ml in whole blood (55, 56). Before Xpert HIV-1 Qual analysis, genomic DNA extracted from 293T cells was RNase treated to eliminate contaminating cellular RNA.

### Western blotting and HIV protein detection.

Total proteins were extracted by direct lysis of samples using RIPA buffer. Extracted proteins were titrated using the Bradford assay (57) and then analyzed by an ELISA or Western blotting. Capsid p24 was measured in the cell extracts and supernatants using a SimpleStep ELISA (Abcam, Cambridge, UK) and an Advia Centaur HIV Ag/Ab combo ELISA (Siemens Healthcare Diagnostics, NY, USA), respectively. RT activity in the supernatants was determined by SG-PERT as described previously by Vermeire et al. (58). Western blot analysis was performed using 20 μg RIPA buffer-extracted proteins developed with either polyclonal human anti-HIV-1 serum (HIV-blot 2.2; MP Diagnostics, Italy) or anti-Tat (catalog number ab42359; Abcam, Cambridge, UK), anti-GFP (catalog number ab183734; Abcam), antiactin (catalog number ab179467; Abcam), or anti-glyceraldehyde-3-phosphate dehydrogenase (GAPDH) (catalog number ab8245; Abcam) antibodies.

### Flow cytometry.

Fluorescent cells were measured using Attune NTX (Thermo Scientific, USA) or FACScan (Becton, Dickinson, Florence, Italy). Cells were analyzed at 2 to 3 days posttransfection or as indicated, following detachment from well plates by trypsin treatment and pelleting by centrifugation at 300 × *g* for 5 min. Data were analyzed using FSC Express 4 software (DeNovo Software, Glendale, CA).

### Statistical analysis.

GraphPad Prism software V5.03 (GraphPad Software, Inc., USA) was used for statistical analysis. Data were analyzed using Student’s *t* test. Differences between groups were considered statistically significant at *P* values of <0.05. All results, including flow cytometry, ddPCR, and *Alu* PCR data, were obtained from at least three independent experiments and are expressed as means ± standard errors of the means (SEM). ddPCR and *Alu* PCR results were analyzed using one-way analysis of variance (ANOVA) (**, *P* < 0.01; ns, not significant). Flow cytometry assay results were analyzed using Student’s *t* test (***, *P* < 0.001; **, *P* < 0.01; *, *P* < 0.1).
